# Regional movements of satellite‐tagged whale sharks *Rhincodon typus* in the Gulf of Aden

**DOI:** 10.1002/ece3.7400

**Published:** 2021-03-23

**Authors:** Samantha Andrzejaczek, Michel Vély, Daniel Jouannet, David Rowat, Sabrina Fossette

**Affiliations:** ^1^ Hopkins Marine Station Stanford University Pacific Grove CA USA; ^2^ Megaptera Paris France; ^3^ Exagone TERIA Vitry‐sur‐Seine France; ^4^ Marine Conservation Society Seychelles Mahe Seychelles; ^5^ Biodiversity and Conservation Science Department of Biodiversity, Conservation and Attractions Kensington WA Australia

**Keywords:** biologging, conservation biology, Djibouti, marine megafauna, migration, telemetry

## Abstract

To gain insight into whale shark (*Rhincodon typus*) movement patterns in the Western Indian Ocean, we deployed eight pop‐up satellite tags at an aggregation site in the Arta Bay region of the Gulf of Tadjoura, Djibouti in the winter months of 2012, 2016, and 2017. Tags revealed movements ranging from local‐scale around the Djibouti aggregation site, regional movements along the coastline of Somaliland, movements north into the Red Sea, and a large‐scale (>1,000 km) movement to the east coast of Somalia, outside of the Gulf of Aden. Vertical movement data revealed high occupation of the top ten meters of the water column, diel vertical movement patterns, and deep diving behavior. Long‐distance movements recorded both here and in previous studies suggest that connectivity between the whale sharks tagged at the Djibouti aggregation and other documented aggregations in the region are likely within annual timeframes. In addition, wide‐ranging movements through multiple nations, as well as the high use of surface waters recorded, likely exposes whale sharks in this region to several anthropogenic threats, including targeted and bycatch fisheries and ship‐strikes. Area‐based management approaches focusing on seasonal hotspots offer a way forward in the conservation of whale sharks in the Western Indian Ocean.

## Graphical abstract

Satellite tags reveal local, regional, and large‐scale movement patterns of eight whale sharks tagged at a Djibouti aggregation site. Long‐distance movements suggest that connectivity exists between this site and other documented aggregations in the region.

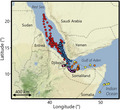

## 1 INTRODUCTION

Marine megafauna are typically highly mobile species with migration pathways that can cross the boundaries of multiple national jurisdictions (Block et al., 2011; Sequeira et al., 2018). These international movements may expose migrating animals to a number of anthropogenic threats, including fluctuating levels of fishing pressure and shipping activity, that together with the varying extent of legal protection encountered through movements and the conservative life histories of many megafauna species, can have large‐scale impacts on populations (Hays et al., 2019). In addition, such movements complicate conservation and management efforts through the need for coordinated efforts among many nations and international organizations (Lascelles et al., 2014). A crucial first step in identifying the threats faced by marine megafauna and in mitigating their potential impacts on populations is to describe the distribution and movement patterns of these vulnerable species (Hammerschlag et al., 2011; Hays et al., 2016).

Whale sharks (*Rhincodon typus*) are large (max. TL 20 m) elasmobranchs that live in warm temperate‐tropical waters (Rowat & Brooks, 2012). In addition to undertaking large‐scale movements (e.g., >7,000 km; Hueter et al., 2013) and crossing international boundaries, these filter‐feeding sharks aggregate seasonally at numerous locations around the world (Sequeira et al., 2013). The reliable aggregation of whale sharks at coastal localities has facilitated the development of an increasingly valuable tourism industry in the past three decades, where divers and snorkelers are able to swim with and observe individuals (Anderson et al., 2014; Gallagher & Hammerschlag, 2011; Zimmerhackel et al., 2019). Despite the successes of these ecotourism ventures (Zimmerhackel et al., 2019), anthropogenic impacts, such as targeted fisheries catches, bycatch in nets, and vessel strikes, continue to jeopardize global whale shark populations (Pierce & Norman, 2016) and, as a consequence, the persistence of this tourism industry. Recent population declines have resulted in the upgrading of whale sharks to globally Endangered on the IUCN Red List of Threatened Species in 2016 (Pierce & Norman, 2016). Global efforts over the past decade to conserve this species have aimed to better understand its movement ecology (Andrzejaczek et al., 2016; McKinney et al., 2017); however, knowledge gaps still remain in understanding the movement patterns of whale sharks after departing their seasonal aggregations.

Photo‐identification (photo‐ID) and satellite tracking are two common approaches to investigating whale shark movements at local to cross‐ocean scales (Andrzejaczek et al., 2016; Berumen et al., 2014; Pierce et al., 2018; Sequeira et al., 2013). Photo‐ID uses unique natural markings to recognize individuals and explore residency and regional movement patterns (Pierce et al., 2018). While this may be an effective technique in regions such as the Western Central Atlantic Ocean where high tourism and directed research efforts exist (McKinney et al., 2017), it may not be viable in areas where direct access to sharks is limited, particularly on remote coastlines and in offshore regions. In addition, these methods are largely limited to when sharks are in surface waters and can lead to misleading conclusions about seasonal habitat use if sharks remain present yet move into deeper, less accessible waters on a seasonal basis (Cagua et al., 2015). Alternatively, satellite tracking is an effective approach that can enable identification of previously undetected habitat hotspots (Diamant et al., 2018; Robinson et al., 2017) and has the added benefit of recording detail about the vertical habitats and thermal environment encountered by a tagged individual.

Numerous aggregations of whale sharks have been identified in the Western Indian Ocean (WIO) including the Maldives, the Gulf of Oman, the Arabian Gulf, Djibouti, the Red Sea, the Seychelles, Tanzania, Mozambique, Madagascar and South Africa (Berumen et al., 2014; Brooks et al., 2010; Diamant et al., 2018; Perry et al., 2018; Pierce & Norman, 2016; Riley et al., 2010; Robinson et al., 2016; Rohner et al., 2015). Despite the remote nature of many of these sites, knowledge of regional movement patterns has been gained through satellite tracking. A total of 131 tracks have so far been collected and published from seven general localities spanning several thousand kilometers (Table 1). These tracks revealed that sharks predominantly remained within the region in which they were tagged; however, many whale sharks were also recorded to cross international boundaries (Berumen et al., 2014; Robinson et al., 2017; Rohner et al., 2018). Increased tagging efforts throughout the WIO are required to continue to build a regional picture of whale shark connectivity within the Indian Ocean and develop management strategies to ensure the long‐term viability of those populations.

**TABLE 1:** Previous satellite tracking studies on whale sharks *Rhincodon typus* in the Western Indian Ocean

Study	Deployment location	Number of successful tags	Duration of deployment
Rowat et al. (2007)	Djibouti	1	9 days
Rowat and Gore (2007)	Seychelles	3	19–60 days
Gifford et al. (2007)	South Africa	3	2–17 days
Brunnschweiler et al. (2009)	Mozambique	2	7–87 days
Berumen et al. (2014)	Red Sea	47	11–315 days
Robinson et al. (2017)	Arabian Gulf	52	1–227 days
Diamant et al. (2018)	Madagascar	8	9–199 days
Rohner et al. (2018)	Mozambique	15	2–88 days

**FIGURE 1:**
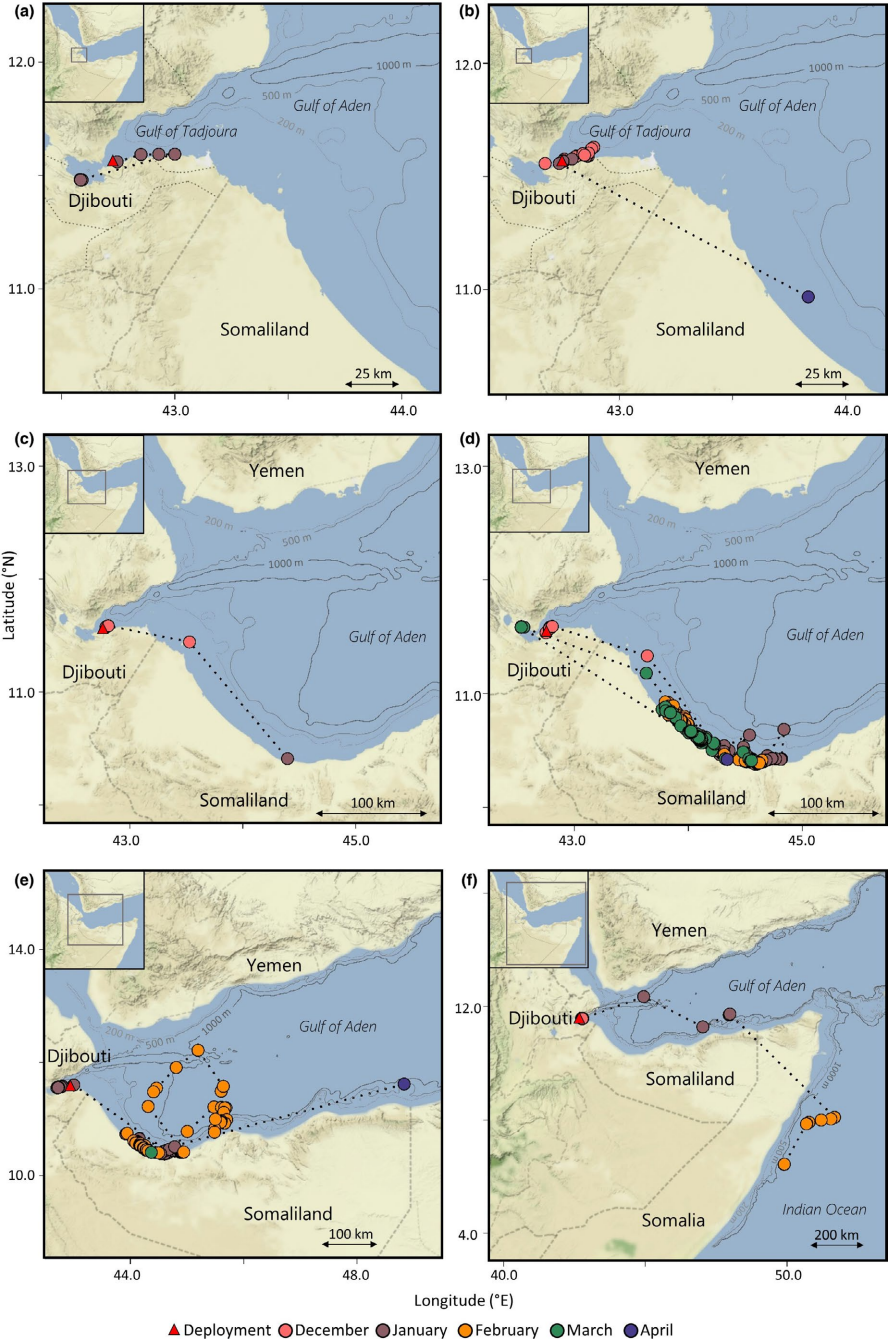
Movements of six whale sharks *Rhincodon typus* tagged with satellite tags in Djibouti in 2016 and 2017. Points indicate location recorded by Argos or GPS and are colored by month. Red triangles indicate tag deployment location. Individuals are (a) 157783; (b) 157782; (c) 42856; (d) 165698; (e) 165699 and; (f) 42858. Note that scale varies among maps. Bathymetry data were extracted from the ETOPO1 database using marmap in R. Dotted lines indicate connection between consecutive points

In the Gulf of Aden, reports of whale shark movement and habitat use have been limited to a seasonal aggregation in the Arta Bay region of the Gulf of Tadjoura, Djibouti (Figure 1). This area is thought to serve as a feeding ground for juvenile whale sharks in the winter months (October to February), where individuals aggregate to forage upon the dense zooplankton and baitfish patches that result from upwelling following the south‐west monsoon (Boldrocchi & Bettinetti, 2019; Boldrocchi et al., 2018; Rowat et al., 2007, 2011). This aggregation was first formally described in 2004 and a photo‐ID study identified upward of 290 individual whale sharks visiting the aggregation between 2003 and 2010, with individuals re‐sighted up to six years after being first recorded there (Rowat et al., 2007, 2011). Individuals were predominately male, and relatively smaller (mean size 3.7 m) than those observed at other aggregations in the Indian Ocean (Rowat et al., 2011). To date, the movement of whale sharks away from the Arta Bay aggregation site has been poorly documented, with records limited to a short (nine day) satellite track from a juvenile individual (Rowat et al., 2007). As this aggregation site is situated close to one of the world's busiest shipping lanes, as well as countries, such as Somalia, with high estimated shark catch through artisanal fisheries (Cashion et al., 2018), these Endangered sharks may be exposed to a myriad of threats while moving throughout the Gulf of Aden. There is therefore a need to further understand the movements and habitat use of whale sharks in Djibouti, and the greater Gulf of Aden region. Given the remote and inaccessible nature of the region surrounding Arta Bay, further electronic tracking offers the best approach to uncovering movement patterns of whale sharks in the Gulf of Aden.

In this study, we describe regional movements and patterns of vertical habitat use of whale sharks on departure from the seasonal aggregation in the Arta Bay region. We discuss the likely drivers of these patterns, the potential for overlap with anthropogenic activities, and the conservation and management implications of our results.

## 2 MATERIALS AND METHODS

### 2.1 Study site and tag deployments

Satellite tags were deployed on whale sharks in the Arta Bay region of the Gulf of Tadjoura (11.57°N, 42.77°E; Figure 1) in January and/or December in 2012 (*n* = 2), 2016 (*n* = 3) and 2017 (*n* = 3; Table 2). Sharks were visually located by boat‐based searches from a 6 m long skiff with a single outboard engine and then approached slowly. Sharks larger than 3.5 m were targeted (a) to satisfy a minimum size for tagging and (b) due to the assumption that only sharks of a certain size migrated from the Gulf of Tadjoura study site. Free‐divers entered the water from the vessel to tag and measure sharks, as well as take photo‐ID images. Tags were deployed by a pole‐spear with a welded plate and rubber buffer to prevent insertion greater than 8–10 cm. 2012 tags were leadered with a ~15 cm length of 45 kg nylon filament covered with several layers of heat shrink tubing and attached via a titanium flat anchor M dart (Wildlife Computers) and placed at the base of the first dorsal fin, on the left side. 2016 and 2017 tags were connected to a large titanium anchor (Wildlife Computers) via a 50 cm stainless steel tether. Tether lengths were selected to allow the anchor to be placed 8–10 cm below the skin and leave space to let the tag lie flat against the body surface in the case of the former, and to facilitate breaking the air‐surface barrier for transmission during deployment for the latter. Individual sharks were measured (total length, TL) by one of two methods; (1) by using visual observation and comparing the shark to an object of known size, and/or (2) by an intense photogrammetric laser measurement campaign using the methods as described in Jeffreys et al. (2013). Photo‐ID images were also taken of the left and right flanks of tagged individuals and matched with the existing Djibouti database using the public domain pattern‐recognition software I^3^S (Interactive Individual Identification System; Van Tienhoven et al., 2007). All fieldwork was approved by and conducted with the knowledge of the Ministry of Environment, Djibouti and local authorities in Arta. All procedures followed standard international guidelines for tagging whale sharks and staff were trained by experts in the field (D. Rowat and M. Meekan; Robinson et al., 2017; Wilson et al., 2006).

**TABLE 2:** Summary details from satellite tag deployments on whale sharks *Rhincodon typus*

Tag ID	Tag type	Sex	TL (m)	Deploy date	Deploy Lat (°N)	Deploy Long (°E)	Pop‐off date	Pop Lat (°N)	Pop Long (°E)	Tracking duration (days)	Transmitting days	Positions per day	Max depth (m)	Geolocation methods
104072	MiniPAT	F	5.0	19 January 2012	11.56	42.76	27 April 2012	16.42	39.51	99	NA	NA	480	I
104073	MiniPAT	M	5.0	19 January 2012	11.56	42.76	28 April 2012	15.96	42.85	100	NA	NA	448	I
157783	MK10	M	4.5	7 January 2016	11.56	42.75	13 January 2016	11.48	42.59	6	5	0.9 ± 0.7 (0–2)	200	A, F
157782	MK10	F	3.5	17 December 2016	11.57	42.76	17 April 2017	10.97	43.83	121	11	0.2 ± 0.9 (0–8)	824	A, F
42856	MK10	M	6.3	17 December 2017	11.58	42.79	2 January 2018	10.41	44.40	16	4	0.5 ± 1.5 (0–6)	NA	A
165699	MK10	M	4.0	22 December 2016	11.57	42.75	1 April 2017	11.62	48.83	100	56	1.8 ± 2.5 (0–12)	1,856	A, F
165698	MK10	M	5.8	18 December 2017	11.57	42.77	2 April 2018	10.42	44.34	105	71	2.0 ± 2.3 (0–10)	272	A, F
42858	MK10	M	4.0	17 December 2017	11.58	42.79	21st February 2018	6.43	49.91	66	14	0.4 ± 0.9 (0–4)	1,344	A, F

Note

All tags were manufactured by Wildlife Computers, Inc. (WA, USA). TL = the total length (m) of the individual tagged estimated by free‐divers. Sex = male (M) or female (F) where determination was possible by visual observation of presence or absence of claspers between the pelvic fins. Pop‐off date = date of tag detachment from shark; Pop Lat/Long = GPS coordinates of tag detachment location; Tracking duration = number of days between tag deployment and pop‐off; Transmitting days = number of days Argos and/or GPS locations were transmitted during deployment; Positions per day = mean ±standard deviation, and range, in number of positions (GPS and/or Argos) transmitted during deployment, Max Depth = the deepest depth (m) reported by the tag during the deployment; and Geolocation Methods = methods used to reconstruct most likely track for each tagged animal: A, Argos location; F, Fastloc GPS; I, Iknos Walker routine.

Pop‐up satellite archival transmitting (PSAT) tags (6 × MK10, 2 × MiniPAT; Wildlife Computers, Inc.) recorded light levels, depth, and ambient temperature and were programmed to remain attached for 100 days (2012), 120 days (2016), or 153 days (2017) or programmed to detach if recording depths greater than 1,800 m or a constant depth reading (±1.0 m) for more than one week. The tags recorded depth and temperature data in predefined bins every six hours for transmission, with depth bin size varying slightly between 2016 and 2017 deployments (0–2, 2–5, 5–10, 10–25, 50–100, 100–200, 200–300, 300–400, 400–500, >500 and 0–10, 10–50, 50–100, 100–250, 250–500, >500, respectively). Histogram sampling was offset by three hours so that depth, and temperature data were collected for local day (6:00–12:00 and 12:00–18:00) and night (18:00–00:00 and 00:00–6:00) periods. The 2012 tags recorded and archived depth data at five‐minute intervals, which were subsequently summarized into bins. A Wilcoxon rank‐sum test was used to compare median day‐ and night‐time depths for time‐series data from the two 2012 tags.

Location data and/or processed archived data were transmitted and retrieved through the Argos satellite system when the tags detached. Detachment of the tag from the shark was identified by a combination of near‐continuous high quality Argos transmissions for the first few hours of each day and depth summaries from histograms consistent with surface records (Hearn et al., 2013). The tags deployed in 2016 and 2017 also transmitted data when sharks swam at the surface, and, in addition, housed a Fastloc global positioning system (GPS) for acquiring location information.

### 2.2 Track reconstruction

A combination of techniques was used to estimate the most probable track for a given individual based on the type and quality of the data transmitted (Table 2).

#### 2.2.1 MiniPAT track reconstruction

The two individuals tagged in 2012 were fitted with MiniPAT tags which did not have the capability to record locations during deployment. Track locations were thus estimated by light‐levels based on data received via Argos transmission after the tags had detached from the host shark; consequently, these were not contiguous data streams. The transmitted data were first processed through the Data Analysis Program software suite (WC‐DAP, Wildlife Computers, Redmond, WA) to extract the dawn/dusk light level as well as the temperature and depth data for each 24‐hr period. These data were further processed through Global Position Estimator suite of programs (WC‐GPE, Wildlife Computers, Redmond, WA) to derive geolocations based on the time of dawn and dusk each day by determining the relationship between the sun's zenith angle and the tag's received light‐level, corrected for any depth attenuation. The date, time, latitude, longitude, estimation error, and sea surface temperature (SST) data were then extracted and formatted into an input file for the Iknos Walker particle filter program (Tremblay et al., 2009) which refines the likely track of the animal by bootstrapping random walks biased by forward particles. The model uses the computed accuracy estimates of the location data and can assimilate other sources of data such as SST and animal speed to further refine the location estimations. The SST recorded by the tags was compared with matching daily remotely sensed geolocated SST data at a grid of 11km^2^, sourced from the NOAA CoastWatch Program, the NOAA NESDIS Office of Satellite Data Processing and Distribution, the NASA’s Goddard Space Flight Centre, and Ocean Color Web (http://coastwatch.pfel.noaa.gov/infog/BA_ssta_las.html). Processing was carried out in the MATLAB numerical computing environment (MATLAB 8.0, The MathWorks, Inc.). The Iknos Walker routine can shorten tracks before the tag detachment location due to the last GPE location(s) being too far away compared with the speed limit set; this can be mitigated by increasing the acceptable animal speed. However, rather than using unlikely speeds, the tracks were re‐run in reverse, starting at the detachment location, and then the two tracks were combined and the most parsimonious daily locations were retained for track output.

#### 2.2.2 MK10 track reconstruction

The six satellite tags deployed on individuals in 2016 and 2017 were programmed to acquire a location estimate from ARGOS and GPS satellites while at the surface. Position estimates acquired from ARGOS satellites were provided with an associated error (Location Class 3: <250 m, 2:250–500 m, 1:500–1,500 m, 0: >1,500 m, A and B: not specified, www.argos‐system.org), and Fastloc GPS positions were expected to have an error of <100 m (Bryant, 2007). For the deployment period, all locations reported from above sea level were removed, as well as a small number of locations with A and B error classes that were obviously erroneous (<1%), that is, they were well beyond the bounds of possible distances the shark could have travelled based on both earlier and later location estimates of higher accuracy for the track. More advanced filtering methods, similar to the ones used for the 2012 tracks, were attempted. However, none of the models converged when using the WC‐GPE3 processor, and the Iknos Walker routine described above could not be applied to the data. These convergence issues were due to large gaps in light, SST, and location data (location and light data available for 9%–68% of tracking days for tracks more than six days in length), as well as the highly coastal nature of the tagged individuals, with locations being classified as “on land” in several cases in the 0.25° grids of GPE3. For the longer tracks, all but one tag displayed transmission gaps >20 days (up to 86 days), preventing unbiased interpolation of tracks between consecutive locations (Queiroz et al., 2016). Attempts were also made to thin known locations from tracks in order to reduce clustering and facilitate model convergence as per Lipscombe et al. (2020); however, resulting track paths diverged significantly from known locations and were deemed unreliable.

## 3 RESULTS

**FIGURE 2:**
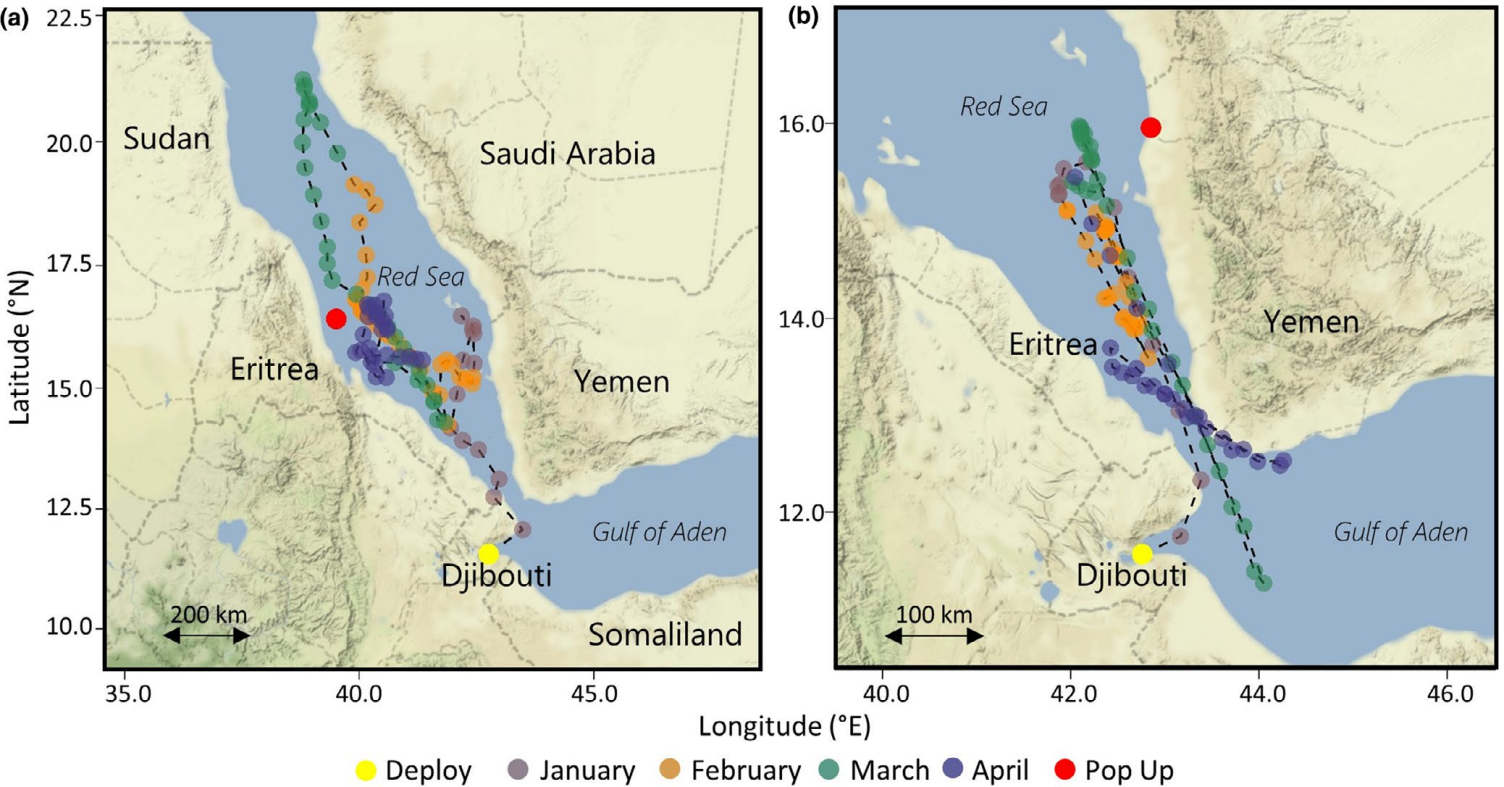
Daily most probable positions of two whale sharks *Rhincodon typus* tagged with satellite tags in Djibouti in 2012. Points indicate location recorded at deployment (yellow), by Argos at pop‐up (red), or by light‐based geolocation (other colors indicating month). Note that pop‐up locations are those first recorded by the tag and may not accurately reflect the real pop‐up location from the tagged individual as a result of delayed data transmissions. Individuals are (a) 104072 and (b) 104073

Eight satellite tags were deployed on eight unique juvenile whale sharks in the Arta Bay region of the Gulf of Tadjoura, Djibouti, in January 2012, January and December 2016 and December 2017 (Table 2). Six males and two females were tagged, and individuals ranged in length 3.5–6.3 m (Table 2). Tags remained attached for between 6 and 116 days (mean ± *SD* =76 ± 42.6 days). For the tags deployed in 2016 and 2017, Argos and/or GPS locations were transmitted on 4 to 71 days of this period (mean ± *SD* =27 ± 29 days; Table 2; Figure 1). For the tags deployed in 2012 which did not have the capacity to transmit locations during deployment, there were discrepancies between the first transmission time after release and depth records, which suggested that the tags may have floated at the surface for some time after release before transmitting location data. As a result, no Argos locations were assigned to the final estimated track end times for these two deployments (Figure 2).

### 3.1 Horizontal movements

Shark movements ranged from local‐scale movements around the Djibouti aggregation site (<100 km), regional movements along the north‐west coastline of Somaliland, a return offshore loop, movements north into the Red Sea, and a large‐scale (>1,000 km) movement to the east coast of Somalia, outside of the Gulf of Aden (Figures 1 and 2). One shark (157783) remained around the Djibouti coast for the entirety of its six‐day track (Figure 1a), while all others departed the Gulf of Tadjoura after varying amounts of time (Figures 1b–f and 2).

Four individuals tracked SE from Djibouti to the NW coastline of Somaliland (Figure 1b–e). For two of these tracks (157782 and 42856), sparse location transmissions (mean ± *SD* positions per day = 0.2 ± 0.9 and 0.5 ± 1.5 respectively) from the tags limited movement inferences following departure from Djibouti to tag pop‐up along the NW Somaliland coast (Figure 1b,c). Shark 157782 remained within the vicinity of the Djibouti tagging location for the first month of tag deployment, after which no locations were transmitted until pop‐up 13 km offshore of NW Somaliland three months later on the 17 April 2017 (Figure 1b). Shark 42856 was tracked within 5 km of the tagging location for the first week of tag deployment before heading SE. The tag for this individual popped up approximately one week later on the 2 January 2018, within 2 km of the coast of NW Somaliland (Figure 1c).

For individuals 165698 and 165699, longer tag attachment durations and more location transmissions enabled detailed descriptions of individual movement around the coast of NW Somaliland (Figure 1d,e). Shark 165698 spent at least three days around the Djibouti aggregation site before reaching NW Somaliland's coast approximately a week later (a straight‐line distance of approximately 220 km; Figure 1d). This individual made relatively consistent movements up and down a 125 km section of coast for almost three months, often remaining within three km of the shore, before making a one‐week return excursion to the Gulf of Tadjoura in late March, traveling at minimum speeds of up to 70 km/day, followed by tag pop‐up within one km of the NW Somaliland coast on the 2 April 2018. Shark 165699 spent at least two weeks around the Djibouti aggregation site before transiting to the NW Somaliland coast (Figure 1e). This individual also made consistent movements up and down the same stretch of coastline as shark 165698 for approximately three weeks, before making a return offshore loop covering at least 625 km into the central Gulf of Aden from 31 January to 14 February 2017, also traveling at minimum speeds of up to 70 km/day. At the northernmost point of this loop, this shark was approximately 160 km north of the coast of Somaliland, and 65 km south of Yemen. On return to the NW Somaliland coastline, shark 165699 spent at least another two weeks moving throughout this area before tag pop‐off 504 km to the NE, three weeks after the last reported transmission (Figure 1e). Following tag pop‐up, location transmissions showed the tag looping around the Gulf of Aden, and arriving in the port city of Little Aden, Yemen, three weeks after pop‐up (Figure S1). For this period, the tag travelled at speeds ranging from 10–50 km/day.

Both sharks tracked in January 2012 (104072 and 104073) moved north into the Red Sea in the week following tag deployment, as indicated by both the most probable tracks and tag pop‐up locations (Figure 2). These individuals moved distances greater than 500 km north from the Djibouti tagging location, with one tag (104072) popping up on the east coast of Eritrea, and the other (104073) on the north‐west coast of Yemen (Figure 2). Shark 104072 remained in the Red Sea from mid‐January to late April and spent more than one month around the coast of Eritrea prior to tag detachment (Figure 2a). Shark 104073 appeared to move between the Red Sea and Gulf of Aden several times before detachment on the Yemen coast (Figure 2b). As no temperature time‐series data were available to match with the depth data, these data stream could not be investigated for fine‐scale changes in the thermal environment as the sharks entered the Red Sea (i.e., Berumen et al., 2014). Profiles of depth and temperature, however, indicated that the temperature during dives to depths greater than 200 m remained above 21°C, supporting presence in the Red Sea (Berumen et al., 2014).

Shark 42858 was the only tagged shark to depart the Gulf of Aden into the Indian Ocean (Figure 1f). Following tagging, this shark spent approximately one week around the Djibouti aggregation site before beginning an offshore migration through the Gulf of Aden, around the NE tip of Somalia, and ultimately heading south along the east coast of Somalia. A detailed insight into this migration is prohibited by limited location transmissions (0.4 ± 0.9 positions per day); however, available data suggest minimum daily speeds of approximately 60–70 km/day and an overall minimum distance of 1,600 km based on straight‐line distances between GPS locations (no Argos positions were available until after pop‐up). Following pop‐up on the 21 February 2018, the tag made directed movements along the shore, reaching a small village along the coast approximately one week later (Figure S1).

### 3.2 Photo‐ID data

Tagged whale sharks were matched with an existing photo‐ID database for the Djibouti aggregation site consisting of 1,039 individuals (D. Rowat unpublished data). Shark 104073 was the only individual observed in subsequent years following tag deployment. It was first recorded in 2007, and observed again each year from 2009 to 2015. For all other sharks, observations were either made in the year of tag deployment only (sharks 157783, 165698, and 165699), or additionally, in the one or two years prior to tag deployment (sharks 104072, 42858, and 157782).

### 3.3 Diving behavior and temperature

**FIGURE 3:**
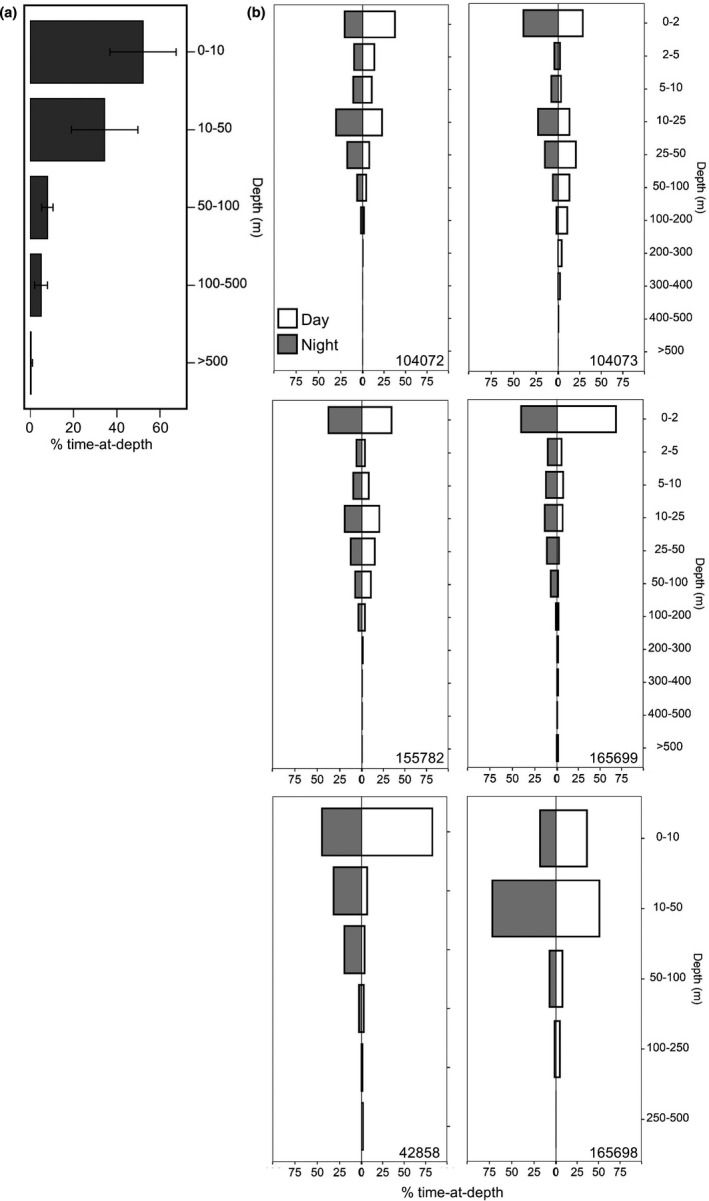
Time‐at‐depth histograms for six whale sharks *Rhincodon typus* tagged with pop‐up satellite archival tags in Djibouti in 2012, 2016 and 2017. (a) Time‐at‐depth for all individuals combined. Error bars represent standard deviation. (b) Diel time‐at‐depth for each individual whale shark. Note that bin width varies between the 2012 and 2016 (top four plots) and 2017 (bottom two plots) deployments

Time‐series and time‐at‐depth histograms (*n* = 368) transmitted from two (2012 tags) and four (2016 and 2017 tags) sharks, respectively, revealed high occupation of the top 50 m of the water column interspersed with occasional deep movements (>200 m) when on offshore excursions, and contrasting patterns of diel vertical movements (DVM). Individuals spent 52.1 ± 15.3% (mean ± *SD*) of the time in the top 10 m, 86.5 ± 4.6% in the top 50 m, and 94.5 ± 2.9% in the top 100 m (Figure 3a), with high individual variation present in the use of the top 50 m (Figure 3b). Higher resolution data from four sharks also revealed high use of the top 2 m (38.36 ± 10.8%; Figure 3b). Maximum recorded depths from seven of the individuals ranged from 200 to 1,864 m (Table 2). Greater than 95% of each track was spent in ambient temperatures of 25–30°C (range 4–30.1°C), with more than 50% of this in 27–29°C (Figure S2).

Individual variation in depth use between coastal and offshore (exceeding the 200 m depth contour) movements was revealed when transmitted depth data could be matched with horizontal locations. When occupying coastal areas of both Djibouti and NW Somaliland, maximum depths ranged from 80 to 200 m, with more than 90% of the time in the top 50 m (*n* = 4 sharks). While within the Gulf of Tadjoura for the month following tag deployment, shark 157782 spent 93% of its time in the top 50 m and was not recorded deeper than 200 m. In the four days leading to tag pop‐off, however, time in the top 50 m reduced to 47% and maximum depths exceeded 300 m daily. In coastal NW Somaliland, shark 165698 spent 40% of its time in the top 10 m, and 97% in the top 50 m while shark 165699 spent 90% of the time in the top 10 m and 99.9% in the top 50 m (Figure 3b). From this coastal spot to the two‐week offshore movement, the use of the top 2 m increased from 20% to 46% for shark 165699, while overall use of the top 50 m decreased to 73%. Notably, 13% of the time was spent at >100 m while this individual was offshore, with depths greater than 1,000 m recorded on six of the 14 days of this return movement as it moved into deeper waters (Figure 1e). Between the last recorded location on the coast to pop‐up, the deepest recorded dive of the study was recorded on the 16 March at 1,864 m, where ambient temperature was 4°C. Lastly, shark 42858 also made several (*n* = 5) dives to greater than 1,000 m in depth while predominately occupying the top 10 m (63% time) as it migrated out of the Gulf of Aden. This individual dove to a maximum depth of 1,344 m while moving along the east coast of Somalia.

Individual whale sharks displayed patterns of either normal DVM (deeper during the day), reverse DVM (deeper at night) or no diel difference in depth distribution (Figure 3). The two sharks that moved into the Red Sea (104072 and 104073) displayed opposite patterns, with shark 104072 moving significantly deeper during the night (median depth = 4.0 m in the day and 13.0 m at night; Wilcoxon rank‐sum test *W* = 1,070.5, *p* < .01) and shark 104073 moving significantly deeper during the day (median depth = 28.0 m in the day and 8.5 m at night; Wilcoxon rank‐sum test *W* = 505, *p* < .05) (Figure 3). Shark 155782 displayed an approximately equal depth distribution between diel periods, and patterns of reverse DVM were recorded by the histogram data for the remaining sharks (165699, 42858, and 165698; Figure 3). Shark 165699 spent 82% of the time in the top 10 m during the day and 64% at night, with this shifting to 58% and 57% for day and night, respectively, during its offshore loop.

## 4 DISCUSSION

Satellite telemetry revealed regional (<500 km) and large‐scale (>500 km) movements through both coastal and offshore habitats of juvenile whale sharks departing the Djibouti aggregation site in the Gulf of Tadjoura. Five of the eight sharks displayed some degree of movement along the coast of Somaliland, with one of these sharks moving out of the Gulf of Aden, and two sharks migrating north into the Red Sea. Depth data revealed high occupation of surface waters, deep diving behaviors, and individual variation in diel vertical movement (DVM) patterns.

### 4.1 Drivers of movement patterns

Following tag deployment in December and January, individual whale sharks remained in the vicinity of the Djibouti aggregation site for up to one month. Previous observations have indicated that these filter‐feeders primarily use this site to forage on the dense aggregations of zooplankton that occur in shallow waters just off the shoreline from October to February (Rezzolla & Storai, 2010; Rowat et al., 2007). Sampling of the surface zooplankton community here has revealed an increasing trend in biomass from November to December, and a decrease from January to February (Boldrocchi et al., 2018), suggesting whale sharks departed as food densities declined. On leaving this site, four of the sharks, tagged across two different years (2016 and 2017), moved south‐east to coastal Somaliland, where two of the longer tracks (one from each 2017 and 2018) revealed sharks patrolling a 125 km stretch of coastline for several weeks. Together with shallow vertical movement patterns, similar to those recorded at the Djibouti aggregation site, such movements recorded in multiple years suggest this may be another whale shark hotspot, with this shark potentially foraging here until at least April (the last month data were received in this study). Validating such a hypothesis, however, is currently not possible given the remote and inaccessible nature of this coastline where there are no dedicated research efforts or tourism. In addition, the relatively short tag attachment period prohibits track descriptions from April to November. It is therefore unknown whether whale sharks remain here or move elsewhere throughout this period, before possibly returning to the Djibouti hotspot, as documented by shark 104073 in 2013, 2014, and 2015, as well as returns by other un‐tagged individuals up to 12 years after first being recorded by photo‐identification records (D. Rowat unpublished data). Notably, no other tagged whale sharks were observed at the Djibouti site in the years after tag deployment. In contrast to the 2016 and 2017 sharks, the two sharks tracked in 2012 moved north into the Red Sea following tag deployment. Such differences in movement patterns between years may reflect recent changes in environmental conditions and therefore reduced habitat suitability in the Red Sea, further illustrated by a reduction in sightings and acoustic detections from tagged sharks in this region in 2017 and 2018, followed by a return to baseline levels in 2019 (Hardenstine et al., 2019). Alternatively, differences in tracked movements could simply represent individual variation. Greater sample sizes and long‐term sighting and tag data paired with environmental data are required to test these hypotheses. The absence of mature animals at the Djibouti site also restricts movement inferences for this population to juvenile individuals. Collectively, annual movement patterns of whale sharks from Djibouti will remain unclear until longer tag attachment durations can be achieved.

Horizontal locations and/or dives into meso‐ or bathypelagic depths indicated that at least five whale sharks moved into offshore habitats over the course of the tracks. Detailed tracks transmitted from two of these sharks (165699 and 42858) revealed both the proportion of time in the top 10 meters and depths greater than 100 m increased during offshore movements, with several dives to more than 1,000 m being recorded. Given available evidence both here and from previous studies, we hypothesize that such vertical behaviors may be driven by foraging at depth, thermoregulation and/or navigation. Shark 165699 showed a two‐week return loop into the Gulf of Aden, which we hypothesize was motivated by foraging. Previous tracking studies have linked deep dives to foraging on meso‐ and bathypelagic layers (Brunnschweiler et al., 2009; Tyminski et al., 2015), and signature fatty acid analysis has suggested that whale sharks from both Ningaloo Reef, Western Australia and Mozambique, attain a significant component of their diet from waters greater than 200 m deep (Marcus et al., 2016; Rohner et al., 2013). As tropical waters tend to be oligotrophic with patchy distributions of prey, declines in prey abundance on the coast may have driven this shark offshore to forage at depth. High use of surface waters (<2 m) may have been a strategy to maintain a preferred body temperature for this ectothermic shark (Thums et al., 2013), following deep dives where ambient temperatures reached a minimum of 4°C at depth. Similarly, the whale shark moving out of the Gulf of Aden also made several dives >1,000 m with high use of the surface ten meters. Alternating between deep and shallow waters may alternatively be a navigational strategy in this case, attaining light and/or celestial cues at the surface (Carey et al., 1990; Lohmann et al., 2008), and magnetic cues by detecting gradients in local field intensity with depth (Klimley, 1993). Such cues have been linked with navigation in migrating white sharks *Carcharodon carcharias*, where tracked sharks primarily occupied the surface two meters of the water column, interspersed with deep dives to >300 m (Bonfil et al., 2010; Weng et al., 2007). Future deployments of high resolution multi‐sensor biologging tags with tri‐axial sensors would provide further insight into the drivers of these vertical movement patterns (Andrzejaczek et al., 2019; Gleiss et al., 2011, 2013).

Inter‐individual variation in DVM patterns may also be a function of the local distribution and behavior of zooplanktonic prey. Of the six individuals with depth data, four recorded patterns of reverse DVM, one of normal DVM, and one with no apparent diel differences in depth use. Notably, opposite patterns were displayed by the two individuals tagged in 2012, with one displaying normal DVM and the other reverse DVM, despite both heading north and into the Red Sea at the same time of year. Additionally, one shark displayed reverse DVM patterns while on the coast, and no diel difference while offshore for two weeks. Reverse DVM patterns have frequently been reported for whale sharks at coastal aggregation sites (Brunnschweiler et al., 2009; Gleiss et al., 2013; Robinson et al., 2017; Rowat & Gore, 2007), with some individuals switching to normal DVM (Brunnschweiler et al., 2009) or no diel pattern (Robinson et al., 2017) on departure from these locations. As DVM patterns have traditionally been explained by the movement of zooplankton to deeper, cooler, and darker waters during the day to reduce detection by visual predators (Hays, 2003), other processes must be driving these reverse behaviors. Local‐scale oceanographic phenomena, for example, could be triggering high productivity and therefore high zooplankton concentrations at the surface during certain diel periods, as is the case for whale sharks foraging in surface waters during the day off the Yucatan Peninsula in Mexico (Motta et al., 2010). Similar to the contrasting patterns recorded in our study, basking sharks (*Cetorhinus maximus*)—another large, filter‐feeding elasmobranch—tracked in the English Channel, displayed patterns of normal DVM in deep, well‐stratified waters and reverse DVM in shallow, inner‐shelf areas near thermal fronts, with such changes in movement related to those of the local zooplankton community (Sims et al., 2005). Alternatively, diel changes in vertical movement patterns of whale sharks may be related to thermoregulatory behaviors (Thums et al., 2013); however, given the lack of temperature change recorded in the coastal habitats occupied here, this seems unlikely.

### 4.2 Connectivity to other known aggregations

**FIGURE 4:**
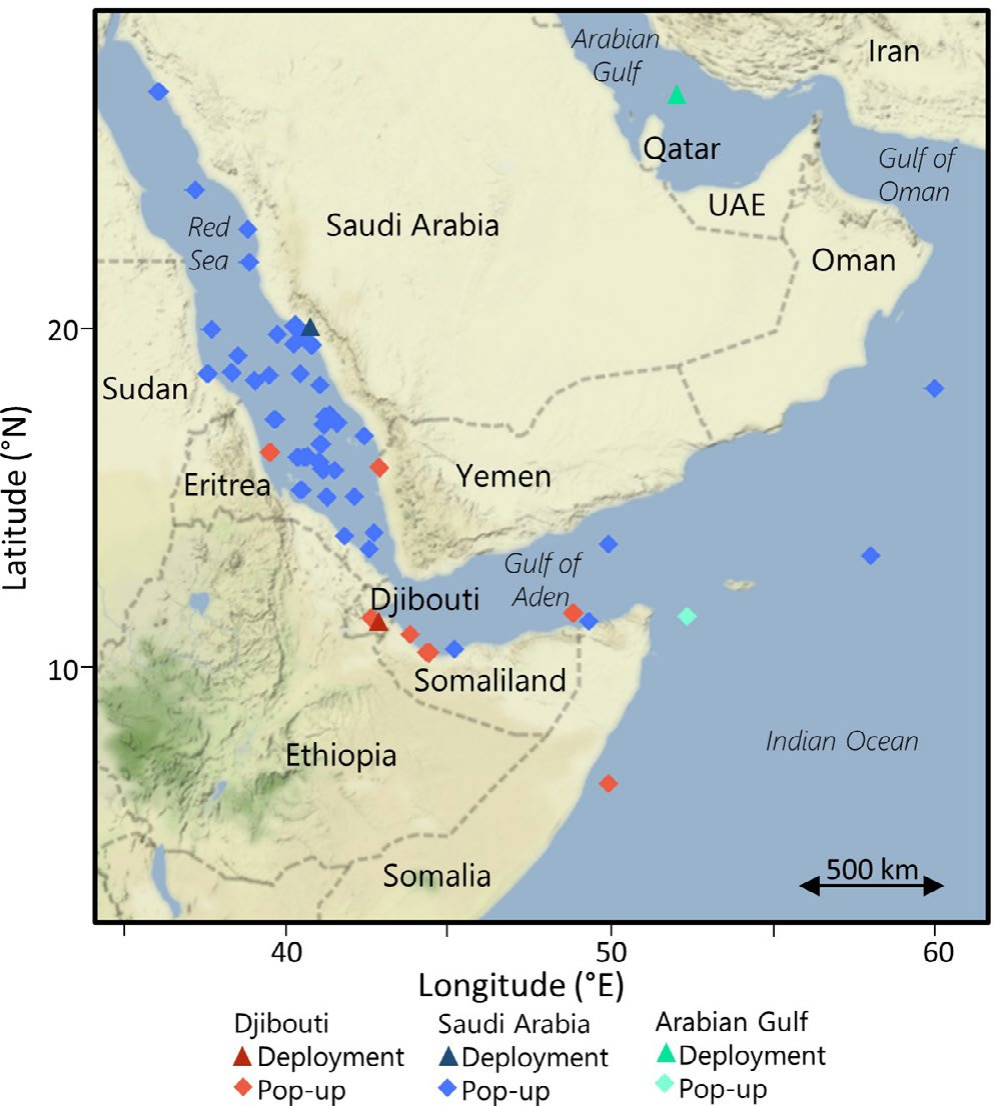
Deployment and pop‐up location(s) from satellite tags deployed on whale sharks *Rhincodon typus* in Djibouti (this study), Saudi Arabia (Berumen et al., 2014), and the Arabian Gulf (Robinson et al., 2017)

Long‐distance movements recorded both here and in previous studies suggest that connections between the whale sharks tagged at the Djibouti aggregation and other documented aggregations are likely. Roughly 10% of the whale sharks tagged along the Saudi Arabian coast between 2009 and 2012 departed the Red Sea into the Gulf of Aden (Figure 4), with one of the tags popping up on the north‐west coast of Somalia (Berumen et al., 2014), close to where at least two of the sharks in this study were tracked. Photo‐identification has also been used to directly link two individual whale sharks to both Djibouti and Saudi Arabia (Norman et al., 2017; D. Rowat unpublished data). Furthermore, the two sharks tagged in Djibouti in 2012 moved north into the Red Sea with tags popping up on the coasts of Yemen and Eritrea. These areas of the southern Red Sea have also been previously reported as areas of high use by whale sharks tagged in Saudi Arabia (Berumen et al., 2014). In addition, the most probable track of shark 104072 displayed a northward movement past the known aggregation site in Saudi Arabia in late February – early March, coinciding with the early aggregation season here (Cochran et al., 2019). In another study, nine sharks tagged in the Arabian Gulf exited into the Gulf of Oman, with one being a large, presumably pregnant, female migrating over 2,644 km south before the tag detached just off the north‐west point of Somalia (Figure 4; Robinson et al., 2017). Seasonal changes in prey availability are thought to be the major driver of movements to and from aggregation sites; however, it is also possible that such long‐distance movements might reflect other aspects of the whale shark's life history (Robinson et al., 2017; Sequeira et al., 2013). For the latter, we are yet to attain satisfactory sample sizes among a representative cross‐section of the population to assess this hypothesis in the Western Indian Ocean. To date, available data from satellite tags deployed in this region have enabled long‐distance movements to be recorded, demonstrating that whale sharks are capable of moving between aggregation sites (Figure 4). Direct evidence of such links, however, has been limited by satellite tag attachment durations. To enhance the probability of recording such links, satellite tags should be deployed toward the end of the peak aggregation season (i.e., February at the Djibouti site) in order to increase attachment time throughout migratory periods (Sequeira et al., 2013).

### 4.3 Management and conservation implications

Whale sharks tracked from the Djibouti aggregation site entered the waters of at least five nations (Djibouti, Yemen, Eritrea, Somalia and Somaliland), where they may face the risks of targeted or incidental capture, entanglement, and boat strike (Lester et al., 2020; Queiroz et al., 2019). The species is afforded varied levels of protection throughout the region, with collective observations and research efforts among these nations highlighting the challenges of implementing and/or enforcing necessary conservation actions. Djibouti is the only nation of the region where whale sharks are protected and are not targeted by fishermen (Berumen et al., 2014; Pierce & Norman, 2016). To the best of our knowledge, no protective measures currently exist in Yemen, Somalia, and Somaliland where active shark fisheries exist that may target these animals (Cashion et al., 2018; D. Obura, personal communication). Additionally, proposed Marine Protected Areas (MPAs) in Djibouti will encompass the largest percentage of the Exclusive Economic Zone (EEZ; 6.2%) relative to the other nations and will include an MPA around the Arta Bay area (IUCN, 2016). In Yemen, MPAs represented 0.5% of the EEZ in 2018; however, given the current political instability in the region, enforcement is unlikely to be held to the usual standards (Z. Samaha, personal communication). In Eritrea, proposed MPA coverage of the EEZ is even lower (0.04%), and, to our knowledge, no proposed or implemented MPAs currently exist in Somalia or Somaliland. These small areas of potential protection, combined with the wide‐ranging movements of tagged individuals on both a horizontal and vertical scale recorded here, suggest whale sharks are at high risk of exposure to anthropogenic threats in the Gulf of Aden and broader region.

The termination of two tracks (165699 and 42858) in urban centers suggests that two sharks tagged in this study may have been caught, further underscoring the need for conservation efforts to be increased in the region. Tracks for these individuals culminated in Aden, a large port city in Yemen, and the other in a village on the east coast of Somalia (Figure 3)—indicating that intentional transportation and landing of this Endangered species may occur in this region. Given rates of tag movement and patterns of surface currents (Zajonz et al., 2016), however, we cannot rule out the possibility that the tags coincidentally drifted into these urban areas, or alternatively that just the tag itself was landed. In either scenario, to improve conservation practices, this whale shark population needs to be managed as a single unit, irrespective of jurisdictional boundaries, throughout its migration cycle (Lascelles et al., 2014). However, as protecting the entire area of whale shark habitat use in the Gulf of Aden region is politically unrealistic and impractical, area‐based conservation approaches focusing on seasonal hotspots and/or regular migratory behavior are likely to be a more constructive way forward (Germanov & Marshall, 2014). Future research should endeavor to refine the current understanding of the patterns of movement of whale sharks throughout their annual migratory cycle in the Gulf of Aden, in order to identify key areas of habitat use and to strengthen the design and implementation of management strategies.

In addition to the threat of capture by fisheries, whale sharks in the Gulf of Aden are also exposed to the threat of ship strike in one of the world's busiest shipping lanes, as well as boat strike by smaller vessels in coastal areas. Similar to results reported elsewhere, whale sharks tracked from Djibouti spent a high proportion of time in surface waters (<2 m) (Motta et al., 2010; Robinson et al., 2017; Thums et al., 2013; Tyminski et al., 2015). At least two individuals tagged in this study made offshore excursions and spent a high proportion of time in surface waters during these ventures. One individual (shark 165699) spent 46% of the time shallower than two meters while in the central Gulf of Aden, increasing its susceptibility to ship strike. Within the Arta Bay region of Djibouti, 15 of 23 individuals observed over a five‐day period had scarring attributable to boat or propeller strikes (Rowat et al., 2007). Management at both domestic and international levels will be required to reduce the impact of these anthropogenic threats and could involve initiatives such as establishing no‐go areas and/or reduced speed limits in important migratory corridors and foraging hotspots and limiting the expansion of marine roads (Pirotta et al., 2019).

## 5 CONCLUSIONS

Our study used satellite tags to reveal patterns of habitat use of whale sharks in the Gulf of Aden, as well as potential connectivity between an aggregation in Djibouti and previously described aggregations in the Western Indian Ocean. The broad horizontal distribution and vertical niche of these sharks in the Gulf of Aden expose them to fishing and shipping activities, threatening the viability of this population. The information collected both here and in previous studies in the region, in combination with continued research efforts, should be used to inform conservation and management strategies at both domestic and international levels.

## CONFLICT OF INTEREST

The authors declare no conflicts of interest.

## AUTHOR CONTRIBUTION

**Samantha Andrzejaczek:** Formal analysis (lead); Investigation (equal); Methodology (supporting); Visualization (lead); Writing‐original draft (lead); Writing‐review & editing (lead). **Michel Vely:** Conceptualization (supporting); Funding acquisition (equal); Investigation (supporting); Project administration (supporting); Writing‐review & editing (supporting). **Daniel Jouannet:** Conceptualization (supporting); Funding acquisition (supporting); Investigation (supporting); Project administration (supporting); Writing‐review & editing (supporting). **David Rowat:** Data curation (supporting); Formal analysis (supporting); Investigation (equal); Methodology (supporting); Writing‐review & editing (supporting). **Sabrina Fossette:** Formal analysis (supporting); Funding acquisition (equal); Investigation (supporting); Methodology (supporting); Project administration (lead); Supervision (lead).

## ETHICS STATEMENT

Fieldwork for this study was conducted in Djibouti with the support and approval of the Ministry of Environment, Djibouti, and local authorities in Arta. All tagging procedures followed standard international guidelines for tagging whale sharks and research staff were trained by experts in the field (Dr Rowat and Dr Mark Meekan).

## Supporting information

Supplementary Material

## Data Availability

Raw tagging data are available from the Dryad Digital Repository: https://doi.org/10.5061/dryad.fqz612js1. Photo‐IDs have been submitted to whaleshark.org.
