# Supplementary material for: Regional movements of satellite‐tagged whale sharks Rhincodon typus in the Gulf of Aden
Source: Ecol Evol. 2021 Mar 23;11(9):4920–34. doi: 10.1002/ece3.7400 (PMC8093710; doi:10.1002/ece3.7400)

Supplementary Information:

Regional movements of satellite-tagged whale sharks Rhincodon typus in the Gulf of Aden

Ecology and Evolution

Samantha Andrzejaczek[1*], Michel Vely[2], Daniel Jouannet[2,3], David Rowat[4] and Sabrina Fossette[2,5]

[1]Hopkins Marine Station, Stanford University, Pacific Grove, CA, USA
[2]Megaptera, 23 rue Alexandre Dumas, Paris, France
[3]Exagone, 29 rue Eugène Derrien 94400 Vitry-sur-Sein, France
[4]Marine Conservation Society Seychelles, Victoria, Seychelles
[5]Biodiversity and Conservation Science – Department of Biodiversity, Conservation and Attractions – Kensington, Western Australia, Australia
*Corresponding author (email: sandrzejaczek@gmail.com)

Supplementary Figure 1. Full tracks, including both Argos and GPS points and movement post pop-up, from tags A) 165699 and B) 42858.

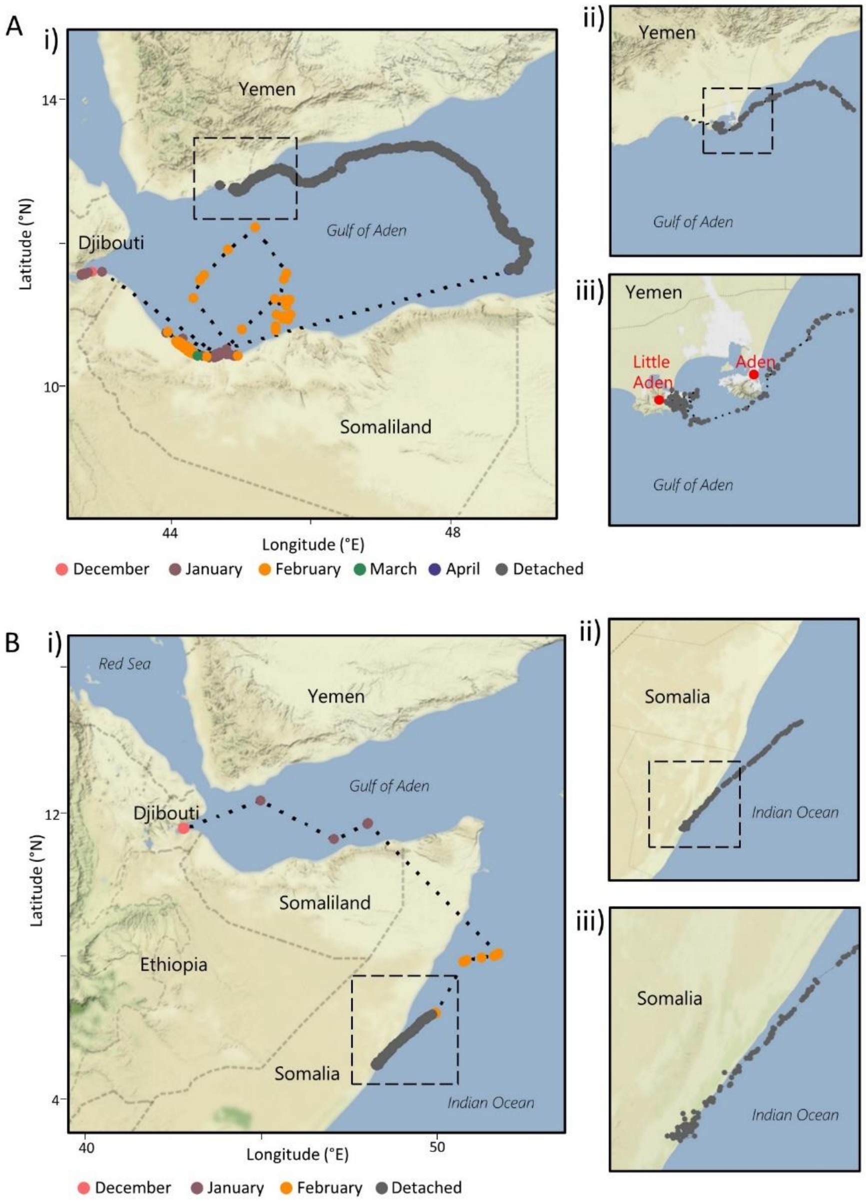

Supplementary Figure 2. Time-at-temperature histograms for six whale sharks Rhincodon typus tagged with pop-up satellite archival tags in Djibouti in 2012, 2016 and 2017. Note that bin width varies between each year of deployment.

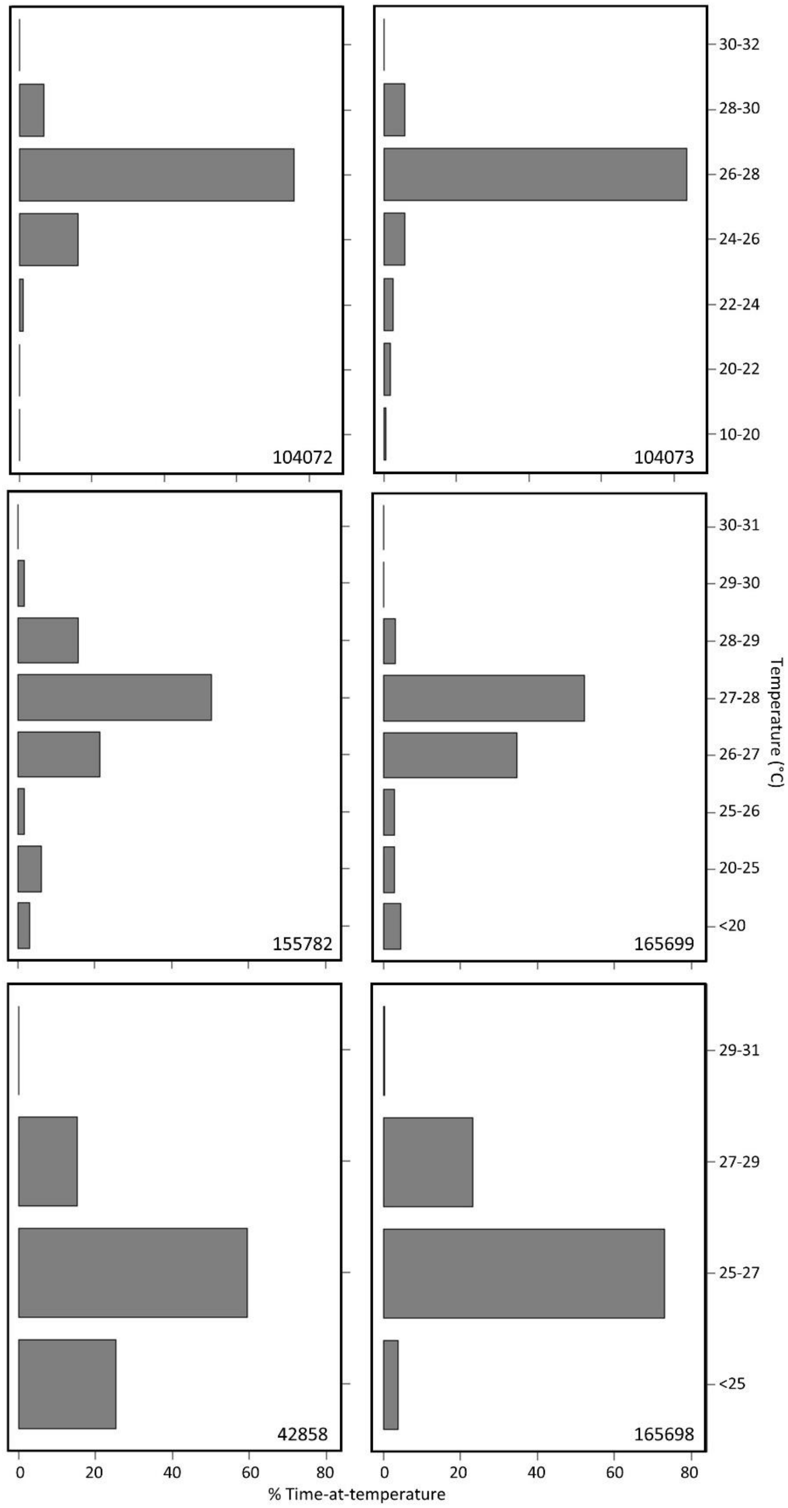

Supplement: Supplementary file 1 — Supplementary Material [file ECE3-11-4920-s001.pdf]
